# Aging Through the Time of COVID-19: Healthcare Access for Older Adults Living With Chronic Conditions

**DOI:** 10.1093/geroni/igab046.1701

**Published:** 2021-12-17

**Authors:** Allie Peckham, Molly Maxfield, Keenan Pituch, M Aaron Guest, Shalini Sivanandam, Brad Doebbeling

**Affiliations:** 1 Arizona State University, Phoenix, Arizona, United States; 4 College of Health Solutions, Arizona State University, Phoenix, Arizona, United States

## Abstract

Chronic conditions require on-going continuous management and preventive treatment. Over 80% of adults aged 65 and older have multiple chronic conditions. Concerns have arisen about how the COVID-19 pandemic is affecting the management of chronic conditions. Delay, avoidance, and poor management of healthcare during the COVID- 19 pandemic may increase the risk of unnecessary hospitalizations and mortality. This study aims to understand the impact of COVID-19 on healthcare access in a U.S. sample of Americans 50 years of age or older. Participants completed an online survey about healthcare access and other risk factors during the COVID-19 pandemic. Multinomial regression analysis examined the results of two key access points: healthcare provider /doctor (n=468) and medication (n=754). One-half (56%) of those who needed access to a provider were able to be seen. Participants who were older, had multiple chronic conditions, and those with a provider were more likely to have access. However, when individuals with more chronic conditions did not have access, they indicated that this lack of access was due to COVID-19. When not receiving access to medications, unemployed participants attributed the lack of access more often to COVID-19 than other reasons. These findings demonstrate an important lack of access to providers and medication among older adults during the pandemic. In multivariate models, this lack of access was most often due to COVID-19, in addition to traditional factors such as insurance, employment, and medical and behavioral comorbidity. Interventions are needed to lower access barriers to care even further during COVID-19.

